# West Nile Virus and Other Domestic Nationally Notifiable Arboviral Diseases — United States, 2020

**DOI:** 10.15585/mmwr.mm7118a3

**Published:** 2022-05-06

**Authors:** Raymond A. Soto, Matthew L. Hughes, J. Erin Staples, Nicole P. Lindsey

**Affiliations:** ^1^Division of Vector-Borne Diseases, National Center for Emerging and Zoonotic Infectious Diseases, CDC; ^2^Epidemic Intelligence Service, CDC.

Arthropod-borne viruses (arboviruses) are transmitted to humans primarily through the bite of infected mosquitoes and ticks. West Nile virus (WNV), mainly transmitted by *Culex* species mosquitos, is the leading cause of domestically acquired arboviral disease in the United States (*1*). Other arboviruses cause sporadic cases of disease and occasional outbreaks. This report summarizes passive data for nationally notifiable domestic arboviruses in the United States reported to CDC for 2020. Forty-four states reported 884 cases of domestic arboviral disease, including those caused by West Nile (731), La Crosse (88), Powassan (21), St. Louis encephalitis (16), eastern equine encephalitis (13), Jamestown Canyon (13), and unspecified California serogroup (*2*) viruses. A total of 559 cases of neuroinvasive WNV disease were reported, for a national incidence of 0.17 cases per 100,000 population. Because arboviral diseases continue to cause serious illness and the locations of outbreaks vary annually, health care providers should consider arboviral infections in patients with aseptic meningitis or encephalitis that occur during periods when ticks and mosquitoes are active, perform recommended diagnostic testing, and promptly report cases to public health authorities to guide prevention strategies and messaging.

Arboviruses are maintained in transmission cycles between arthropods and vertebrate hosts, including humans and other animals. In the United States, humans primarily become infected when bitten by an infected mosquito or tick and rarely through other routes such as blood transfusion and organ transplantation. Most human infections are asymptomatic; symptomatic infections commonly manifest as systemic febrile illness and less commonly as neuroinvasive disease.

Most endemic arboviral diseases are nationally notifiable and are reported by state health departments to CDC through ArboNET, the national arboviral disease surveillance system, using standard surveillance case definitions.* Cases are reported by a patient’s state and county of residence. Confirmed and probable cases with onset of illness during 2020 are included in this report. Cases with reported meningitis, encephalitis, acute flaccid paralysis, or unspecified neurologic signs or symptoms were classified as neuroinvasive disease; other cases were classified as nonneuroinvasive disease. Incidence was calculated using 2020 midyear population estimates from the U.S. Census Bureau.^†^ Incidence calculations were limited to neuroinvasive disease; these cases are more consistently diagnosed and reported because of disease severity. This activity was reviewed by CDC and was conducted consistent with applicable federal law and CDC policy.^§^

A total of 884 cases of domestic arboviral disease were reported for 2020. Cases were caused by the following viruses: West Nile (731, 83%), La Crosse (88, 10%), Powassan (21, 2%), St. Louis encephalitis (16, 2%), eastern equine encephalitis (13, 1%), Jamestown Canyon (13, 1%), and unspecified California serogroup (two, <1%; exact virus unknown). Cases were reported from 306 (10%) of the 3,143 U.S. counties. No cases were reported from Alaska, Delaware, the District of Columbia, Hawaii, Minnesota, Rhode Island, or Vermont.

Cases of WNV disease were reported from 226 counties in 40 states; 83% of patients had illness onset during July–September (Table 1). The median patient age was 62 years (IQR = 50–71 years); 459 (63%) were male. A total of 583 (80%) patients were hospitalized, and 66 (9%) died. The median age of patients who died from neuroinvasive disease was 70 years (IQR = 64–82 years).

**TABLE 1:** Number and percentage of reported cases of West Nile virus and other arboviral diseases (N = 884), by virus type and selected patient characteristics — United States, 2020

Characteristic	Virus type,* no. (%) of cases					
	West Nile (n = 731)	La Crosse (n = 88)	Powassan (n = 21)	St. Louis encephalitis (n = 16)	Eastern equine encephalitis (n = 13)	Jamestown Canyon (n = 13)
**Age group, yrs**						
<18	16 (2)	83 (94)	3 (14)	0 (—)	3 (23)	0 (—)
18–59	302 (41)	3 (3)	6 (29)	5 (31)	2 (15)	6 (46)
≥60	413 (56)	2 (2)	12 (57)	11 (69)	8 (62)	7 (54)
**Sex**						
Male	459 (63)	52 (59)	14 (67)	12 (75)	7 (54)	10 (77)
Female	272 (37)	36 (41)	7 (33)	4 (25)	6 (46)	3 (23)
**Period of illness onset**						
Jan–Mar	5 (1)	1 (1)	1 (5)	1 (6)	0 (—)	0 (—)
Apr–Jun	38 (5)	7 (8)	10 (48)	2 (13)	0 (—)	7 (54)
Jul–Sep	607 (83)	76 (86)	4 (19)	10 (63)	12 (92)	6 (46)
Oct–Dec	81 (11)	4 (5)	6 (29)	3 (19)	1 (8)	0 (—)
**Clinical syndrome**						
Nonneuroinvasive	172 (24)	4 (5)	1 (5)	2 (13)	0 (—)	3 (23)
Neuroinvasive	559 (76)	84 (95)	20 (95)	14 (88)	13 (100)	10 (77)
Encephalitis^†^	343 (61)	66 (79)	13 (65)	8 (57)	12 (92)	9 (90)
Meningitis^†^	150 (27)	16 (19)	5 (25)	3 (21)	1 (8)	0 (—)
AFP^†,§^	26 (5)	0 (—)	1 (5)	0 (—)	0 (—)	0 (—)
Unspecified^†^	40 (7)	2 (2)	1 (5)	3 (21)	0 (—)	1 (10)
**Outcome**						
Hospitalization	583 (80)	83 (94)	17 (81)	15 (94)	13 (100)	10 (77)
Death	66 (9)	0 (—)	1 (5)	3 (19)	4 (31)	0 (—)

**Abbreviation:** AFP = acute flaccid paralysis.

* Two unspecified California serogroup virus cases were also reported.

^†^ Percentages of cases of encephalitis, meningitis, AFP, and unspecified neurologic signs or symptoms are percentages of neuroinvasive cases.

^§^ Among the 26 West Nile virus disease cases with AFP, six persons (23%) also had encephalitis or meningitis.

In 2020, a total of 559 cases of neuroinvasive WNV disease were reported. The national incidence of neuroinvasive WNV disease was 0.17 cases per 100,000 population (Table 2). The highest incidences occurred in South Dakota (1.12 per 100,000) and Nebraska (0.46) (Figure). The largest numbers of neuroinvasive cases were reported from California (179), Texas (101), Florida (44), and Illinois (36), which together accounted for 64% of cases nationwide. Sixteen counties accounted for approximately 50% of WNV neuroinvasive disease cases. Incidence of neuroinvasive WNV disease increased with age, from 0.01 per 100,000 among children aged <10 years to 0.49 among adults aged ≥70 years. Incidence was higher among males (0.22) than among females (0.12).

**TABLE 2:** Number and rate* of reported cases of neuroinvasive arboviral disease, by virus type, U.S. Census Bureau division, and state — United States, 2020^†^

U.S. Census Bureau division/State	Cases, by virus type, no. (rate)*					
	West Nile	La Crosse	Powassan	St. Louis encephalitis	Eastern equine encephalitis	Jamestown Canyon
**United States**	**559 (0.17)**	**84 (0.03)**	**20 (0.01)**	**14 (<0.01)**	**13 (<0.01)**	**10 (<0.01)**
**New England**	**15 (0.10)**	**—^†^**	**9 (0.06)**	**—**	**5 (0.03)**	3 (0.02)
Connecticut	5 (0.14)	—	2 (0.06)	—	—	—
Maine	1 (0.07)	—	1 (0.07)	—	—	—
Massachusetts	9 (0.13)	—	6 (0.09)	—	5 (0.07)	—
New Hampshire	—	—	—	—	—	3 (0.22)
Rhode Island	—	—	—	—	—	—
Vermont	—	—	—	—	—	—
**Middle Atlantic**	**28 (0.07)**	**—**	**7 (0.02)**	**—**	**—**	**—**
New Jersey	3 (0.03)	—	1 (0.01)	—	—	—
New York	18 (0.09)	—	2 (0.01)	—	—	—
Pennsylvania	7 (0.05)	—	4 (0.03)	—	—	—
**East North Central**	**76 (0.16)**	**34 (0.07)**	**4 (0.01)**	**—**	**7 (0.01)**	**7 (0.01)**
Illinois	36 (0.29)	1 (0.01)	—	—	—	—
Indiana	3 (0.04)	1 (0.01)	—	—	1 (0.01)	—
Michigan	29 (0.29)	—	—	—	4 (0.04)	3 (0.03)
Ohio	3 (0.03)	30 (0.26)	—	—	—	—
Wisconsin	5 (0.09)	2 (0.03)	4 (0.07)	—	2 (0.03)	4 (0.07)
**West North Central**	**30 (0.14)**	**—**	**—**	**—**	**—**	**—**
Iowa	2 (0.06)	—	—	—	—	—
Kansas	6 (0.21)	—	—	—	—	—
Minnesota	—	—	—	—	—	—
Missouri	1 (0.02)	—	—	—	—	—
Nebraska	9 (0.46)	—	—	—	—	—
North Dakota	2 (0.26)	—	—	—	—	—
South Dakota	10 (1.12)	—	—	—	—	—
**South Atlantic**	**58 (0.09)**	**30 (0.05)**	**—**	**—**	**1 (<0.01)**	**—**
Delaware	—	—	—	—	—	—
District of Columbia	—	—	—	—	—	—
Florida	44 (0.20)	—	—	—	—	—
Georgia	7 (0.07)	1 (0.01)	—	—	—	—
Maryland	1 (0.02)	—	—	—	—	—
North Carolina	1 (0.01)	21 (0.20)	—	—	—	—
South Carolina	4 (0.08)	1 (0.02)	—	—	1 (0.02)	—
Virginia	1 (0.01)	1 (0.01)	—	—	—	—
West Virginia	—	6 (0.34)	—	—	—	—
**East South Central**	**11 (0.06)**	**20 (0.10)**	**—**	**—**	**—**	**—**
Alabama	7 (0.14)	—	—	—	—	—
Kentucky	—	5 (0.11)	—	—	—	—
Mississippi	4 (0.13)	—	—	—	—	—
Tennessee	—	15 (0.22)	—	—	—	—
**West South Central**	**122 (0.30)**	**—**	**—**	**3 (0.01)**	**—**	**—**
Arkansas	1 (0.03)	—	—	—	—	—
Louisiana	14 (0.30)	—	—	—	—	—
Oklahoma	6 (0.15)	—	—	—	—	—
Texas	101 (0.34)	—	—	3 (0.01)	—	—
**Mountain**	**40 (0.16)**	**—**	**—**	**6 (0.02)**	**—**	**—**
Arizona	8 (0.11)	—	—	6 (0.08)	—	—
Colorado	17 (0.29)	—	—	—	—	—
Idaho	5 (0.27)	—	—	—	—	—
Montana	1 (0.09)	—	—	—	—	—
Nevada	—	—	—	—	—	—
New Mexico	7 (0.33)	—	—	—	—	—
Utah	1 (0.03)	—	—	—	—	—
Wyoming	1 (0.17)	—	—	—	—	—
**Pacific**	**179 (0.33)**	**—**	**—**	**5 (0.01)**	**—**	**—**
Alaska	—	—	—	—	—	—
California	179 (0.45)	—	—	5 (0.01)	—	—
Hawaii	—	—	—	—	—	—
Oregon	—	—	—	—	—	—
Washington	—	—	—	—	—	—

* Cases per 100,000 population, based on July 1, 2020, U.S. Census Bureau population estimates.

^†^ Dashes indicate no cases reported.

**FIGURE:**
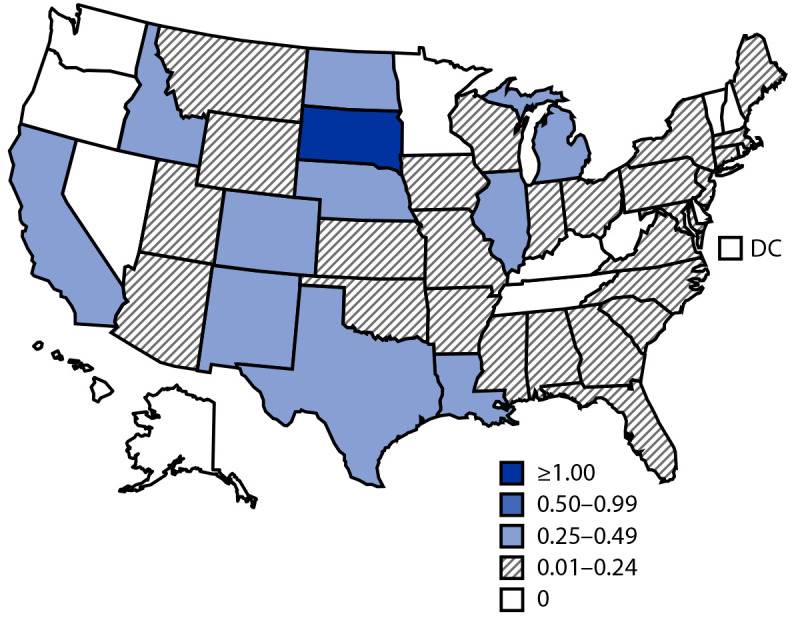
Incidence* of reported cases of neuroinvasive West Nile virus disease — United States, 2020 **Abbreviation:** DC = District of Columbia. * Cases per 100,000 population, based on July 1, 2020, U.S. Census Bureau population estimates.

Eleven states reported 88 La Crosse virus disease cases, with the highest numbers from Ohio (33) and North Carolina (21). The median patient age was 7 years (IQR = 4–11 years); 83 (94%) were children aged <18 years (Table 1). Most patients (86%) had illness onset during July–September. Eighty-four (95%) patients had neuroinvasive disease and 83 (94%) were hospitalized; no deaths were reported.

Twenty-one cases of Powassan virus disease were reported from seven states, with the highest number (seven, 33%) from Massachusetts. The median patient age was 64 years (IQR = 45–69 years); 14 (67%) were male (Table 1). Dates of illness onset ranged from March to November, with 10 (48%) occurring during April–June. Twenty (95%) patients had neuroinvasive disease, and 17 (81%) were hospitalized. One patient, aged >60 years, died.

Sixteen cases of St. Louis encephalitis virus disease were reported from four states (Arizona, California, Pennsylvania, and Texas). The median patient age was 71 years (IQR = 58–80 years); 12 (75%) were male (Table 1). Ten patients (63%) had illness onset during July–September. Fourteen (88%) patients had neuroinvasive disease, and 15 (94%) were hospitalized. Three (19%) deaths were reported, all among patients aged >60 years.

Thirteen cases of eastern equine encephalitis virus disease were reported from five states (Indiana, Massachusetts, Michigan, South Carolina, and Wisconsin); all were classified as neuroinvasive disease. The median patient age was 61 years (IQR = 44–62 years); seven patients (54%) were male. Most patients (92%) had illness onset during July–September. All patients were hospitalized, and four (31%) died; among the fatal cases, three were aged >50 years and one was aged <18 years.

Thirteen Jamestown Canyon virus disease cases were reported from three states (Michigan, New Hampshire, and Wisconsin). The median patient age was 60 years (IQR = 54–69 years); 10 (77%) were male (Table 1). Illness onset dates ranged from April to September, with 54% occurring during April–June. Ten (77%) patients had neuroinvasive disease and 10 (77%) were hospitalized; no deaths were reported.

## Discussion

WNV was the most common cause of domestic arboviral neuroinvasive disease in the United States during 2020. La Crosse virus continued to be the most common cause of neuroinvasive arboviral disease in children; eastern equine encephalitis virus remained the arboviral disease most likely to result in death, with 31% of reported cases being fatal (*1*).

The annual incidence of WNV neuroinvasive disease in 2020 (0.17 per 100,000) was the lowest since 2011 (0.16 per 100,000) and 59% lower than the median annual incidence during 2010–2019 (0.41; range = 0.16–0.92) (*1*,*2*). Areas with the largest decreases in neuroinvasive disease incidence in 2020 compared with the previous decade were the West North Central, East South Central, and Mountain regions. Arizona reported the lowest total number of cases (12) since WNV was first detected in the state in 2003. Despite the relatively low annual incidence, WNV caused most neuroinvasive arboviral disease cases in 2020.

Although neuroinvasive WNV disease incidence was low in 2020, national incidences for all other domestic arboviral diseases were higher than the median annual incidences during the previous decade; increases ranged from 22% for La Crosse virus to 133% for St. Louis encephalitis virus (*3*–*7*). The number of La Crosse virus disease cases reported was the highest since 2011 (*3*). Compared with 2019, fewer cases of eastern equine encephalitis virus disease (13 versus 38) and Powassan virus disease (21 versus 39) were reported, but national incidence for each arbovirus disease was still higher than average for the preceding 10 years (*4*,*5*).

Although persons reported spending more time outdoors during the coronavirus disease 2019 (COVID-19) pandemic, thus increasing potential exposure to arboviral diseases, changes in health care seeking–behavior, prioritization of diagnostic testing for SARS-CoV-2 (the virus that causes COVID-19), and challenges in reporting of arboviral disease cases concurrent with COVID-19 likely affected reporting of arboviral disease cases, particularly for nonneuroinvasive disease cases (*8*,*9*). The percentage of WNV cases classified as neuroinvasive disease (76%) was higher than the average reported during 2010–2019 (59%), suggesting that less severe disease cases were less likely to be diagnosed and reported during the COVID-19 pandemic. The number of jurisdictions reporting arboviral disease cases (44) was the lowest since 2014, which suggests that public health capacity to investigate and report cases also might have been affected by the pandemic. However, because the annual incidence of arboviral diseases varies based on weather, zoonotic host factors, vector abundance, and human behavior, gauging the actual impact of COVID-19 on the occurrence, recognition, and reporting of arboviral diseases is challenging.

The findings in this report are subject to at least two limitations. First, ArboNET is a passive surveillance system that leads to underestimation of actual disease prevalence. For a case to be captured by the system, a patient must seek care, the clinician must request appropriate diagnostic tests, and results must be reported to public health authorities. An estimated 30–70 nonneuroinvasive cases occur for every neuroinvasive WNV disease case reported (*10*). On the basis of the number of neuroinvasive WNV disease cases reported for 2020, from 16,770 to 39,130 nonneuroinvasive WNV disease cases are estimated to have occurred; only 172 (≤1%) were reported. Second, because ArboNET does not require information about clinical signs, symptoms, or laboratory findings, clinical syndrome of certain cases might be misclassified.

Understanding the epidemiology, seasonality, and geographic distribution of arboviruses is important for clinical recognition. Arboviral diseases continue to cause serious illness and the locations of outbreaks vary annually. Therefore, health care providers should consider arboviral infections in patients with aseptic meningitis or encephalitis that occur during periods when ticks and mosquitoes are active, perform recommended diagnostic testing, and promptly report cases to public health authorities to guide prevention strategies and messaging. Because predicting locations and timing of arboviral disease cases is difficult, timely surveillance remains critical to identifying outbreaks and guiding prevention efforts. Prevention depends on community and individual efforts to reduce vector populations,^¶^ personal protective measures to decrease mosquito** and tick^††^ exposures, and implementation of blood donation screening to minimize transfusion transmission.^§§^

**Summary**

**What is already known about this topic?**

West Nile virus is the leading cause of domestically acquired arboviral disease. Other arboviruses cause sporadic cases and outbreaks, resulting in substantial morbidity and mortality.

**What is added by this report?**

In 2020, the national incidence of neuroinvasive West Nile virus disease was 59% lower than the median annual incidence during 2010–2019. However, the neuroinvasive disease incidence for other domestic arboviral diseases was higher in 2020 than the median annual incidence for the preceding 10 years.

**What are the implications for public health practice?**

Health care providers should consider arboviral infections in patients with aseptic meningitis or encephalitis during periods when mosquitoes and ticks are active, perform recommended diagnostic testing, and promptly report cases to public health authorities to guide prevention strategies and messaging.

## Footnotes

*
https://ndc.services.cdc.gov/case-definitions/arboviral-diseases-neuroinvasive-and-non-neuroinvasive-2015/

†
https://www.census.gov/programs-surveys/popest.html

§ 45 C.F.R. part 46, 21 C.F.R. part 56; 42 U.S.C. Sect. 241(d); 5 U.S.C. Sect. 552a; 44 U.S.C. Sect. 3501 et seq.

¶
https://www.cdc.gov/mosquitoes/mosquito-control/index.html

**
https://www.cdc.gov/mosquitoes/mosquito-bites/prevent-mosquito-bites.html

††
https://www.cdc.gov/ticks/tickbornediseases/tick-bites-prevention.html

§§
https://www.cdc.gov/bloodsafety/basics.html
